# Lethal chickenpox infection during treatment with infliximab and methotrexate

**DOI:** 10.1186/1546-0096-9-S1-P226

**Published:** 2011-09-14

**Authors:** A Estmann, P Toftedal, AJ Schou

**Affiliations:** 1 Hans Christian Andersen Children’s Hospital, Odense, Denmark

## Background

Using immunosuppressive drugs in younger children increases the risk of severe infections not seen in adult patients, as immunity towards several viruses is lacking.

## Aim

To increase the awareness of the risk of severe infections in patients without immunity towards several viruses.

## Methods

In a seven-year-old boy treatment with methotrexate and infliximab for bilateral panuveitis complicated by glaucoma and cataract was initiated. During treatment the uveitis went into remission and oral treatment with prednisolone could be stopped. The intraocular pressure dropped and surgical intervention for glaucoma was cancelled. The child underwent screening for tuberculosis before treatment was started. Also, as the child had not received any vaccinations in his entire life, vaccination against measles, rubella and mumps (part of the normal Danish vaccination strategy of children) was performed before treatment.

After four months of treatment the boy showed signs of chickenpox and was admitted to hospital due to viral pneumonia. The immunosuppressive drugs were withdrawn and antiviral treatment initiated immediately in high doses. He developed progressively respiratory insufficiency and required mechanical ventilation and as the condition worsened Extracorporeal Membrane Oxygenation (ECMO). He finally died from increased intracranial pressure due to encephalitis.

## Conclusion

Before initiating treatment with immunosuppressive drugs it is of severe importance to evaluate the immunity of the patient towards viruses able of causing severe infection. Vaccination prior to treatment must be considered.

